# Operational Parameters for Sub-Nano Tesla Field Resolution of PHMR Sensors in Harsh Environments

**DOI:** 10.3390/s21206891

**Published:** 2021-10-18

**Authors:** Taehyeong Jeon, Proloy Taran Das, Mijin Kim, Changyeop Jeon, Byeonghwa Lim, Ivan Soldatov, CheolGi Kim

**Affiliations:** 1 Department of Emerging Materials Science, DGIST, Daegu 42988, Korea; wonil075201@dgist.ac.kr (T.J.); kmj@dgist.ac.kr (M.K.); jco4268@dgist.ac.kr (C.J.); 2 Magnetics Initiative Life Care Research Center, DGIST, Daegu 42988, Korea; limbh@dgist.ac.kr; 3 Leibniz Institute for Solid State and Materials Research (IFW) Dresden, Helmholtzstraße 20, D-01069 Dresden, Germany; i.soldatov@ifw-dresden.de; 4 Institute of Natural Sciences and Mathematics, Ural Federal University, 620075 Yekaterinburg, Russia

**Keywords:** magnetoresistive sensors, planar-Hall magnetoresistance, sensitivity, field detectivity

## Abstract

The resolution of planar-Hall magnetoresistive (PHMR) sensors was investigated in the frequency range from 0.5 Hz to 200 Hz in terms of its sensitivity, average noise level, and detectivity. Analysis of the sensor sensitivity and voltage noise response was performed by varying operational parameters such as sensor geometrical architectures, sensor configurations, sensing currents, and temperature. All the measurements of PHMR sensors were carried out under both constant current (CC) and constant voltage (CV) modes. In the present study, Barkhausen noise was revealed in *1/f* noise component and found less significant in the PHMR sensor configuration. Under measured noise spectral density at optimized conditions, the best magnetic field detectivity was achieved better than 550 pT/√Hz at 100 Hz and close to 1.1 nT/√Hz at 10 Hz for a tri-layer multi-ring PHMR sensor in an unshielded environment. Furthermore, the promising feasibility and possible routes for further improvement of the sensor resolution are discussed.

## 1. Introduction

Extremely-low magnetic field sensing in the low-frequency range is an important task for a wide range of application areas such as magnetic random access memory (MRAM) [1], neuromorphic computing [2], magnetic communication [3,4], noninvasive biomedical diagnosis [5,6,7,8], nondestructive materials evaluation [9], human–machine interaction [10,11], robotics [12], and automotive or consumer-based industrials [13]. Most of these applications demand miniaturization, low-cost fabrication, high thermal stability, flexibility in usage, and robustness in a harsh environment with a very high sensor resolution [14,15,16,17]. In this perspective, many researchers have been involved in the development of an ultra-sensitive magnetic sensor over the past ten years, and it has been found that magnetoresistive (MR) sensor is always one of the best choices due to its high scalability and inherent capability to install ROIC (readout integration circuit) and also due to its excellent integration compatibility with CMOS (complementary metal-oxide-semiconductor) MEMS (micro-electro-mechanical systems) devices [18,19,20,21]. Especially, with the development of MEMS and nanofabrication technologies, integration of MR sensors into high-density chips also makes it possible to realize multiplex detection and to develop point-of-care detection, minimizing device size [22,23]. The most challenging task in MR sensing technology is to overcome low-frequency noise below 100 Hz, which affects the critical field detection limit of the valuable frequency range in modern biomedical and industrial applications. Thus, there are still many open questions regarding *1/f* noise characteristics, which limit the high signal-to-noise ratio required for low-frequency applications below 10 Hz. Different strategies have been demonstrated to reduce the low-frequency noise in MR sensors [24,25,26,27] by integrating magnetic flux concentrators [14,15,20], implementing hybrid magnetic-MEMS devices [28], magnetic tunnel junction stacks [29], or large sensor arrays [30,31].

Note that sensor sensitivity and noise levels are also affected by environmental conditions such as temperature, shielding, etc.; however, the effect of temperature on the total average noise of the MR sensors remains unexplored. Temperature dependence of the noise power spectral density and its detailed systematic analysis in MR sensors always provides a promising directive for its application even in harsh environments. In the literature, most of the noise analysis of MR sensors [16,21,32,33,34,35,36] has been done by comparing the experimental noise spectra with different numerical models, and the analyses deal with sensor noise performance at room temperature only, whereas very few systematic works have been carried out to investigate the total average noise as a function of temperature [37]. In the temperature dependence of the noise spectrum, an additional noise component appears due to the rise of temperature, generally caused by thermal drift. Thus, it is a more challenging task to retain the noise at the same level with the increase in temperatures. In addition, the electrical and magnetic characterization of these MR sensors is a prerequisite for use in applications, especially when small magnetic fields and related field amplitude changes are involved. Thus, it is important to study sensitivity, drift behavior, and noise properties, including their temperature dependence. It can be envisaged that noise measurement under different operational conditions, such as various temperatures and/or sensing currents, might add some salient features in the sensor characterization. Moreover, it can be helpful for understanding the origins and manifestations of various noise sources, which may lead to improvement in sensor robustness and the development of novel applications [38]. Recently, we decomposed noise source components of different planar-Hall magnetoresistive (PHMR) sensors in order to address their high thermal stability and low temperature drift characteristics [17]. Earlier studies reveal that because of their field-dependent sensitivity improvement [37] and unique self-balanced noise compensating feature [21], PHMR sensors exhibit several advantages as compared with other MR sensors [39,40,41]. Moreover, these unique features favor improving the sensor detection limit in a low-frequency regime.

Here, we report the temperature-dependent sensitivity and field-detectivity performance of PHMR sensors, configured as a bi-layered cross junction (bi-cPHMR), a bi-layered multi-ring (bi-mPHMR), and a tri-layered multi-ring (tri-mPHMR), in the temperature range of 30–90 °C. Here, sensor thermal stability is also examined by adjusting the temperature dependences of the exchange coupling and anisotropy. In order to improve the sensor resolution in a low-frequency regime, several physical parameters were optimized, such as sensing current dependency in constant current mode (CC) and constant voltage (CV) mode, temperature variation, and different architectural geometries. Note that for each noise measurement, the recorded noise spectrum was guided by an analytical noise power spectral density (NSD) fit model as proposed by Grosz et al. [42,43].

In this paper, we first discuss the theoretical background for the relationship of operational parameters and follow by a description of the experimental setups for sensor magnetic, electronic, and noise characterizations. Next, we report sensor performance in the framework of several operational parameters, which include temperature, sensing current dependencies of sensor sensitivity, offset voltage variations, and the time evolution of domain wall (DW) propagation, in addition to magnetic NSDs in terms of thermal noise, environmental noise, intermixing noise background, and Barkhausen noise. Finally, sensor detectivity (D) is evaluated for different sensing currents and temperatures, and an outline of possible future work is presented.

## 2. Background for the Relationship of Operational Parameters

Field resolution is generally referred to as the detectivity of the sensor [44,45]. With the operational parameters of electrical sensing input of the sensor, it is required to evaluate the temperature dependence of total noise, $V_{noise}^{total}$, and the sensor’s field sensitivity, *S*, to analyze the detectivity as follows:

(1) $$D\left(T,I,V\right)=\frac{V_{noise}^{total}\left(T,I,V\right)}{S\left(T,I,V\right)}\;\;\;\;\;\;\left[\frac{T}{\sqrt{Hz}}\right]$$

**Figure 1:**
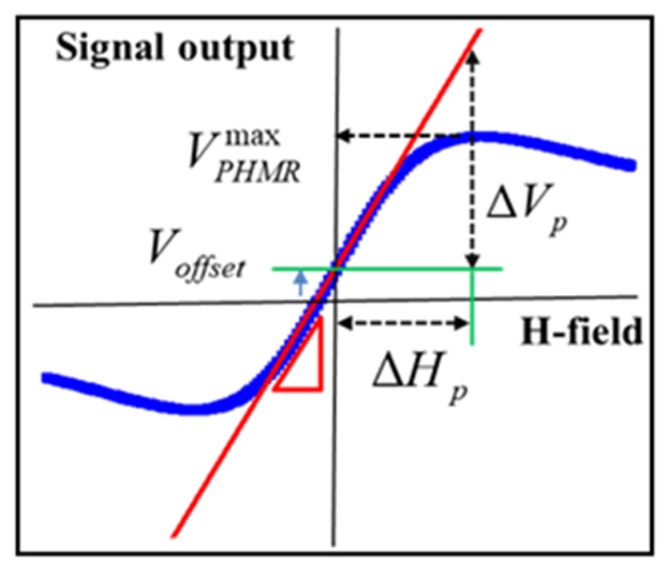
Schematic representation of sensitivity estimation from a PHMR signal profile.

In order to improve the detectivity, it is essential to increase *S* and/or to reduce the overall noise of the sensor. In fact, the sensor’s field sensitivity corresponds to the change in signal response with respect to the external field. As for a typical PHMR signal, as shown in Figure 1, the average field sensitivity can be expressed as [46]

(2) $$S=\mu_{0}^{-1}{\frac{\Delta V_{p}\;\left(H,T\right)}{\Delta H_{p}}|}_{\;}$$

where $\Delta V_{p}$ is the extrapolated peak voltage related to maximum voltage $\Delta V_{PHMR}^{max}\;$ at peak field, and $\Delta H_{p}$ is the field interval from zero to maximum voltage field. The $\Delta V_{PHMR}\;$ depends on several operational parameters such as sensor geometry (cross and ring-type), sensing current density, temperature, etc. Because the sensor resistance changes with temperatures with its coefficient in the order of 10^−4^ °C^−1^ [17], net current density, which causes the planar-Hall effect, is dependent on the operational parameters of constant current (CC) and constant voltage (CV) modes. In *CC mode*, the net current density is kept constant, while in *CV mode*, the net current density is reduced due to the increase in resistance with increasing temperature. Thus, the PHMR response ($\Delta V_{PHMR}$) is related to field dependence of change in the off-diagonal resistance component, $\Delta R_{xy}$, in *CC mode*, while in *CV mode* it depends on the ratio between $\Delta R_{xy}$ and the resistance, $R_{o}$, that is associated with electrical sensing voltage terminals, which can be written as follows:

(3)ΔVPHMR(H,T)≈ΔRxy(T,H)Ix+Voffset(I, T)  (CC mode)(4)≈ΔRxy(T,H)Ro(T)Vx+Voffset(V,T)(CV mode)

where $I_{x}\;\mathrm{and}\;V_{x}\;$ refer to the sensing current and voltage along with the sensor easy axis. Here, $V_{offset}$ refers to the sensor offset voltage. Owing to the characteristics of the planar-Hall effect, $V_{offset}$ should be “zero” for perfect cross-type and balanced PHMR sensor configuration. However, due to the imperfection of sensor fabrication, $V_{offset}$ is not “zero”.

The peak field, Δ*Hp* is governed by the internal field of the sensing layer of NiFe in multi-layer structure. In exchange biased sensing layer with antiferromagnetic layer, IrMn, the local field interval is given by the sum of exchange coupling field $H_{ex}$ and magnetic anisotropy field $H_{a}\;$ of the sensor element as follows [47]:

(5) $$\Delta H_{p}\left(T\right)=\sqrt{\frac{2}{3}}(H_{ex}\left(T\right)+H_{a}\left(T\right))\;\;\;$$

The $H_{ex}$ is ~13 and 3 mT for the bilayer and tri-layers structure [48], respectively. Thus, the $H_{ex}\;$ is the dominant contribution to the internal field for bilayer structure because the anisotropy of NiFe is around ~0.5 mT [49]. Even in tri-layers structure, $H_{ex\;}$ is more than 4 times larger than the anisotropy field. However, it is not easy to evaluate its contribution to the internal field interval of a micrometer-sized sensor. It is worthwhile to note that both $H_{ex}$ and $H_{a\;}$ decrease with the temperature irrespective of bilayer and tri-layers structures. Based on the PHMR voltage and local field interval of Equations (3)–(5), the field sensitivity can be rewritten as follows:

(6)S(T)≈(ΔRxy(T, H)Is)/ΔHp(T)   (CC mode),(7)≈ (ΔRxy(T, H)Ro(T)Vx)ΔHp(T)(CV mode)

Here, it is noted that temperature dependence of sensitivity refers to the ratio of the off-diagonal resistance and internal field variation with temperature in *CC mode*, while in *CV mode*, it is defined as the ratio between normalized off-diagonal resistance component ($\frac{\Delta R_{xy}\left(T,\;H\right)}{R_{o}\left(T\right)})$ and the internal field variation as a function of temperature.

As the second parameter of detectivity, total noise of the PHMR sensor ($V_{noise}^{total}$) in low-frequency generally receives contributions from three major components, referred to as internal noise ($V_{int})$, external noise ($V_{ext})$, and intermixing noise ($V_{mix})$, and the low-frequency noise model of PHMR sensors can be framed as follows [21]:
$\left\{ \begin{array}{l} V_{noise}^{total}=\sqrt{{V_{noise}^{2}|}_{int}+{V_{noise}^{2}|}_{ext}+{V_{noise}^{2}|}_{mix}}\;\;\;\;\;\;\left[\frac{V}{\sqrt{Hz}}\right]\;\mathrm{with},\\ {V_{noise}^{2}|}_{int}=V_{offset}^{2}\frac{\delta_{H}}{n_{c}\cdot \;Vol\cdot \;f^{\alpha }}+{\;V_{noise}^{2}|}_{thermal}+{V_{noise}^{2}|}_{MBN}\\ {V_{noise}^{2}|}_{ext}={\;V_{noise}^{2}|}_{amp}+\;\;{V_{noise}^{2}|}_{env}\\ {V_{noise}^{2}|}_{mix}=\;V_{offset}^{2}\left(\frac{\delta I_{n}}{I_{x}}\right) \end{array}\right.$(8)(9)(10)(11)

In the intrinsic noise portion, the first term is the pink noise (*1/f*) contribution [43,45,50] containing the offset voltage $V_{offset}$, charge carrier density of Ni_80_Fe_20_, and *n_C_* = 17 × 10^28^/m^3^ [51], assuming that the major contribution of the sensing current was passed through the FM layer [52], the Hooge’s constant $\delta_{H}$ [53], and the fit parameter α. Vol refers to the total effective volume for the PHMR sensor, and it is evident that Vol is inversely proportional to the total *1/f* noise and that it becomes dominant in small sensing areas. Note that for the exchange-coupled sensors, $\delta_{H}$ usually varies at an order of 10^−2^ to 10^−3^; however, the value of $\delta_{H}$ can be higher due to the presence of magnetic fluctuations [45,54].

In case of an MR sensor, the thermal noise is given by Johnson–Nyquist noise level [21,50,55]:

(12) $${V_{noise}^{2}|}_{thermal}=4k_{B}TR_{yy}$$

with the sensor transverse resistance, $R_{yy}\;\mathrm{of}\;\mathrm{voltage}\;\mathrm{measuring}\;\mathrm{electrodes},$ temperature, and the Boltzmann’s constant, *k_B_*. Moreover, the MR sensors also exhibit magnetic Barkhausen noise (*MBN*) signals in the low-frequency range and can be expressed as [56]

(13) $${V_{noise}^{2}|}_{MBN}=\beta \frac{\partial M}{\partial H}\left(H\right)$$

where *M* is the magnetization within the sensor (M–magnetization, $\frac{\partial M}{\partial H}$–magnetization fluctuations), and β refers to a constant.

External noise is composed of the noise of the amplifier and the environmental noise ${V_{noise}^{2}|}_{env}$ portion. In addition, intermixing noise will also appear due to additional voltage offset, $V_{offset}$, which occurs along the transverse to sense current direction because of a fabrication mismatch of the PHMR electrode. $\delta I_{n}$ is referred to as the Nyquist noise of the operating current from the power source [21]. In order to evaluate the detectivity for the optimal operational parameters, it is required to characterize the temperature dependence of the PHMR signal, the sensor’s field sensitivity, *S*, and the drift of the offset voltage in CC and CV modes, in addition to decomposed noise, $V_{noise}^{total}$ including *MBN*.

## 3. Materials and Methods

### 3.1. Sensor Fabrication

**Figure 2:**
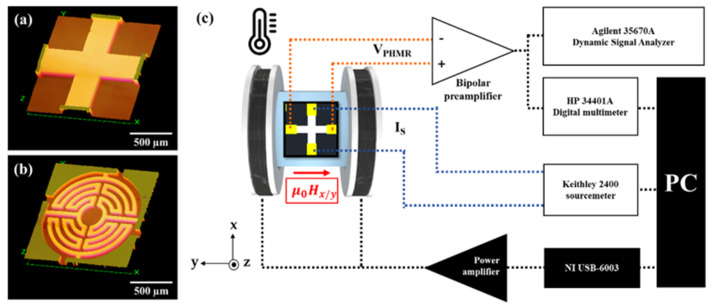
Optical microscopic images of (**a**) bilayer cross-type PHMR (bi-cPHMR) and (**b**) multi-ring type bi(tri) layer PHMR (mPHMR) sensor. (**c**) Schematic of the noise measurement setup, including electrical connection and the external field application $\left(\mu_{0}H_{x,y}\right)$.

In this study, three different types of sensors i.e., bi-cPHMR, bi-mPHMR, and tri-mPHMR were fabricated using UV lithography and DC-magnetron sputtering system. For mPHMR sensors, the Wheatstone bridge multi-ring geometry concept was used [57], and the original number of rings with the designed standard ring pattern was set to five. A detailed description of sensor fabrication technology can be found elsewhere [21]. The sensing area for the bi-cPHMR sensor was ~90,000 μm^2^, and the corresponding value for mPHMR sensors was ~427,835 μm^2^. Bi-layer structures were made of Ta (5 nm)/NiFe (10 nm)/IrMn (10 nm)/Ta (5 nm) and grown on a 500 nm SiO_2_ substrate by wet oxidation process, whereas for tri-layer structure, the sensor structure was Ta (5 nm)/NiFe (10 nm)/Cu (0.5 nm)/IrMn (10 nm)/Ta (5 nm) and grown on the similar SiO_2_ substrate. Figure 2a,b shows optical microscopic images of the bi-cPHMR and mPHMR sensor geometries. The sensor layers were capped with a thin Ta layer (5 nm) to prevent oxidation. The samples were etched such that the magnetic easy axis coincided with the sensing current direction (along the *x*-axis). The chosen dimensions were determined according to the capability of fabricating optimized PHMR sensors [40] without any detrimental imperfections.

### 3.2. Sensor Characterization

Sensor sensitivity was determined by applying a dc magnetic field along the *y*-axis ranging from −3.5 to 3.5 mT using a Helmholtz coil set up (see Figure 2c). A standard F.W. Bell 5080 gaussmeter was used for the magnetic field estimation and placed in close vicinity to the sample position. In order to investigate the thermal stability and temperature dependence of the sensor sensitivity, a water-cooled hot plate, working in the 30–90 °C temperature range, was installed surrounding the sample mounting position [17]. Once the temperature was set and stabilized within the limit of ±0.1 °C (at least for 15 min), the PHMR signals were recorded. Note that these temperature-dependent sensitivity measurements, S(T), were carried out in both CC and CV modes. In both cases, the sensitivity was derived from the slope of linear response in its dynamic range and the applied field, as demonstrated in Equations (6) and (7), respectively. Furthermore, sensor sensitivities were studied for different sensing currents ranging from 1 to 7 mA. A Keithley 2400 current SourceMeter and an HP 34401A voltmeter were used to generate the sensor sensing current and to record the PHMR signal, respectively. The measurements were performed in an unshielded environment and repeated a number of times in the same experimental protocol for better precision.

### 3.3. Noise Measurements

For noise characterization, the sensor current was applied along the magnetic easy axis using a Keithley 2400 Current Source Meter. The sensor signal was amplified by a custom-made low-noise preamplifier [58] and sampled by a 16-bit ADC model (35670A Keysight FFT Dynamic signal analyzer) with a sampling rate of 512 Hz. Temperature dependence of noise measurements was performed in both CC and CV modes at different sensing currents. A schematic of the measuring system is shown in Figure 2c.

In addition, magneto-optical Kerr effect microscopy (MOKE) [59,60] was utilized for the simultaneous measurement of the domain evolution. MOKE hysteresis loops and domain images were captured in a Zeiss wide-field polarization microscope with a combined optical path. The entire system was equipped with an in-plane standard electromagnet and light source suitable for selective sensitivity in Kerr microscopy [61,62].

## 4. Results and Discussion

### 4.1. Temperature Dependence of PHMR Signals

**Figure 3:**
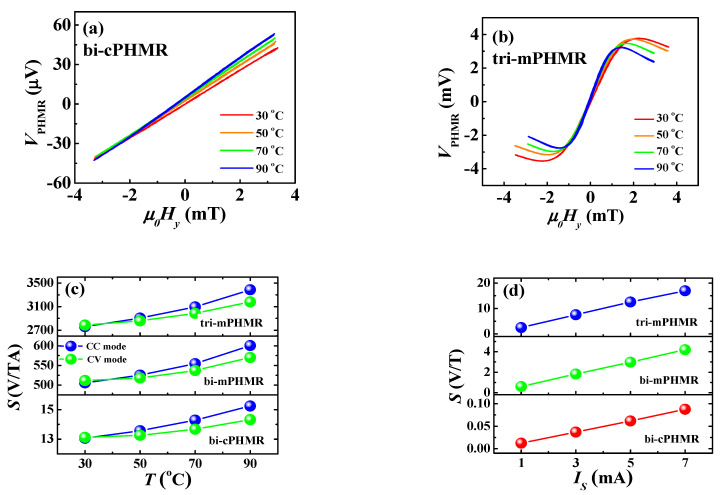
(**a**,**b**) Change in PHMR voltage with temperatures in CC mode for bi-cPHMR and tri-mPHMR sensors; (**c**) sensitivity change with temperature and applied sensing current for 1 mA; (**d**) sensing current dependence on sensitivity.

**Figure A1:**
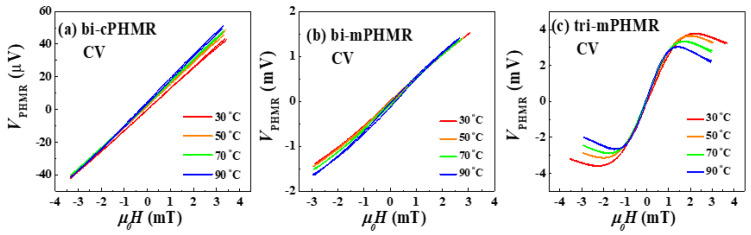
(**a**–**c**) Variation of amplified PHMR voltages with temperature for different sensors in CV mode.

The output voltage of the PHMR sensors was measured as a function of the magnetic field in CC and CV modes at a sensing current of 1 mA as well as measured for various temperatures ranging from 30 to 90 °C (see Figure 3a, band Figure A1 in Appendix A section). It is important to note that the V_PHMR_–H curves at different temperatures show a negligible difference between each other, suggesting that the behavior of the PHMR within its operating dynamic range was extremely stable within the ~90 °C temperature range in both modes. Figure 3a demonstrates the temperature dependence of the PHMR signal for bi-cPHMR sensor measured in CC mode, governed by Equation (3). Detailed temperature dependence of PHMR responses for the bi-cPHMR sensor can be found in our earlier report [17], thus omitted here. In experimental configurations, an external magnetic field, H_y_, was applied transverse to the easy magnetization axis (see Figure 2c) of the sensors. It was found that for the bi-cPHMR sensor, the sensitivity increased monotonically with temperature within the applied magnetic field range of ±3.5 mT; however, no signature of maximum V_PHMR_ was observed in the field range smaller than the exchange field strength of 13 mT. Importantly, in this case, the recorded V_PHMR_ exhibited similar behavior for all temperatures. Figure 3a reveals that the PHMR amplitude of the bi-cPHMR sensor decreased with the increase in temperature, although the estimated change in the offset voltage was minimal. Generally, the reduction in PHMR signal amplitudes with temperature occurred because of the diminution of the anisotropic resistivity Δρ with Kohler’s rule [63]. A maximum of 16% diminution in PHMR amplitude was derived at 90 °C compared with its value at room temperature.

Similar PHMR signals were obtained for other multi-ring PHMR sensors. Note that, like the bi-cPHMR sensor, no signature of peak-to-peak V_PHMR_ output was found for the bi-mPHMR sensor within the ±2 mT field range, which was smaller than H_ex_ (see Figure A1b); however, the tri-layer structure exhibited the full curve of V_peak-to-peak_ response in PHMR voltage for all temperatures where the estimated *H_ex_* was ≈3 mT, as shown in Figure 3b. Generally, PHMR sensors exhibit maximum planar-Hall response when the angle, θ, between sensing current direction and sensor magnetization was equal to $\frac{\pi }{4}$. Beyond that, a shallow reduction in PHMR voltage is observed as it varies with sin2θ . It is evident from the figures that, in the tri-mPHMR sensor, the operating dynamic field range was reduced due to the decrease in the internal field of *H_ex_* and *H_a_* in Equation (5); however, the relative reduction in dynamic field range with the increase in temperature was found to be similar for all PHMR sensors.

Moreover, Figure 3b demonstrates that the response curves for forward and backward sweeping almost completely overlapped with each other. No hysteresis was observed up to 70 °C, and only at 90 °C the full field range sweeping shows a negligible hysteresis. Importantly, in our earlier reports [21,38], no signature of signal hysteresis was observed even in the higher temperature for single ring-PHMR and cross-PHMR sensors, which might be due to the decrease in exchange coupling at higher temperatures ≥90 °C [64]. Furthermore, similar characterizations were performed in CV mode for all sensors, and no unusual behavior was observed in sensor response (see Figure A1 in Appendix A). Based on the experimental results, we observed a slightly better sensor response in CC mode compared with its CV counterpart. Note that, a broad dynamic range was found for all PHMR sensors, which demonstrates promising sensing applications in higher temperature regimes.

### 4.2. Temperature Dependence of Field Sensitivity

Figure 3c exhibits the temperature dependence of field sensitivity response of PHMR sensors, which is basically governed by the ratio of temperature-dependent PHMR voltage and internal field, as in Equations (6) and (7), respectively. The sensing current was set to 1 mA. In each case, the sensor sensitivity demonstrated a nonlinear behavior with temperature. The maximum sensitivity was found in the tri-mPHMR sensor, and the estimated value was 3386 V/TA at 90 °C, whereas for the other two sensors the estimated values were 601 V/TA (bi-mPHMR) and 15 V/TA (bi-cPHMR). The observed results indicate that the tri-mPHMR sensor demonstrated higher sensitivity due to the lower exchange coupling field compared with the other two PHMR sensors, which makes it more promising for ultra-low magnetic field detection. The highest increment in sensitivity for the tri-mPHMR sensor was ~22.7% at 90 °C, whereas for other sensors, the observed change was around 16.7% (bi-cPHMR) and 18.8% (bi-mPHMR). The results confirm that the maximum sensitivity growth was observed for the mPHMR sensor, and moreover, the sensitivity in CC mode grew faster than sensitivity in CV mode, becoming 6.8% higher at 90 °C (see Figure 3c), which means that the ratio $\frac{\Delta R\left(T\right)}{\Delta H_{p}\left(T\right)}$ was more constant than the ratio, $\frac{\left(\frac{\Delta R\left(T\right)}{R\left(T\right)}\right)}{\Delta H_{p}\left(T\right)}$.

Figure 3d demonstrates a rapid and linear increment in the sensor sensitivity, with an increase in sensing current at room temperature. The field sensitivity was increased by a factor of ~6.8, 6.9, and 7.1 for tri-mPHMR, bi-mPHMR, and bi-cPHMR sensors, respectively. The maximum sensitivity estimated for the tri-mPHMR was ~17 V/T at 7 mA. However, at high temperatures, it was difficult to pass through a high sensing current due to an increase in thermal noise and the occurrence of other technical constraints (discussed in Section 4.4.3). The substantial enhancement in sensitivity at high sensing current at room temperature refers to an increase in MR in these sensing devices [65,66]. In CV mode, the increment in sensitivity for different sensing voltages was found to be similar. Based on the presented results, we optimized the sensing current to 7 mA in CC mode. Note that due to the detrimental thermal effect at higher temperatures, the optimized current was set to ~3 mA for temperature-dependence studies.

In order to obtain a high field sensitivity, it is required to enhance the PHMR voltage response amplitude, which is determined by the percentage of active current passed through the active layer and/or to decrease the extremum field (exchange coupling field). An optimized tri-mPHMR structure [67,68] is capable of allowing high active current to pass through the active layer and also possesses small exchange coupling in the system. These are prerequisite requirements for achieving high field sensitivity in the sensor. The results presented here confirm that the tri-mPHMR sensor was a more desirable candidate for obtaining high field sensitivity compared with the other two bi-layered structures. Moreover, the obtained results confirm that a sensor’s geometrical architecture, along with a sensor’s sensing of current/voltages, plays a crucial role in the improvement of sensor sensitivity [17,65].

### 4.3. Temperature Dependence of Offset Drifts

**Figure 4:**
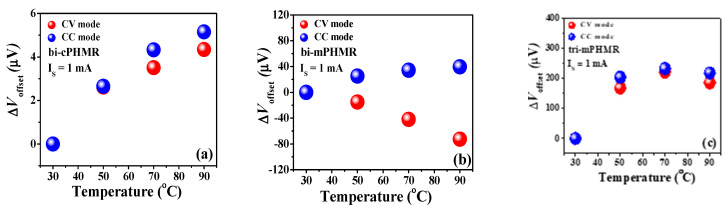
(**a**–**c**) Baseline offset drifts (ΔV_Baseline_) with temperature change in CC and CV modes.

The change in the drift of baseline as a function of temperature for bi-cPHMR, bi-mPHMR, and tri-mPHMR sensors was measured using the same experimental setup as shown in Figure 2c, which is basically caused by the fabrication imperfection as in Equations (3) and (4). Nonlinear temperature dependence in baseline drift was observed in each sensor, as depicted in Figure 4. In order to investigate how the sensor drift behavior in CC and CV mode depended on temperature, all sensors were measured multiple times in both modes. From the experimental data, it was seen that independent of the mode, the sensor baseline drifts shifted to the higher side with an increase in temperature. However, at the highest temperature, a slight downturn was observed for all sensors (except the CV mode characteristic of the bi-mPHMR sensor). The change in drifts in mPHMR sensors was more pronounced in CV mode.

**Figure 5:**
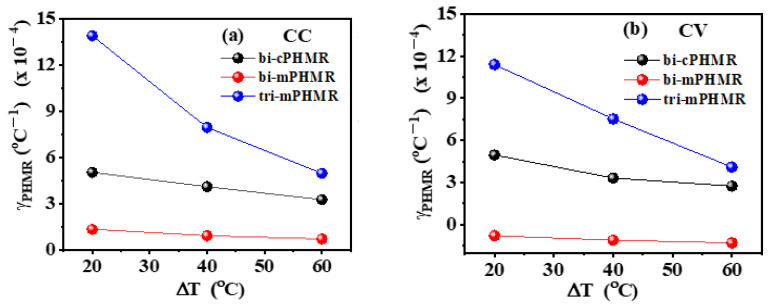
(**a**,**b**) Temperature-dependent variation of $\gamma_{PHMR}$ in CC and CV modes. ΔT refers to the relative temperature increment with respect to room temperature (30 °C).

**Figure 6:**
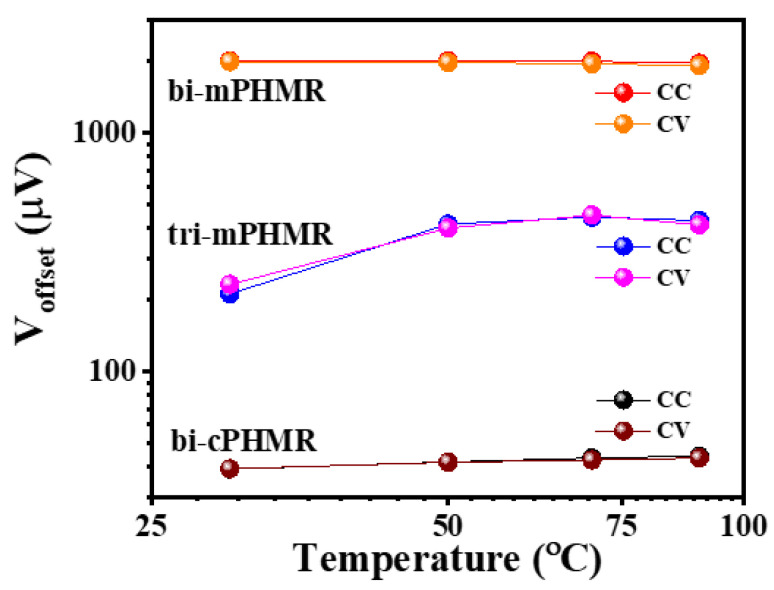
Sensors offset voltage variation with temperatures. Sensing current was 1 mA.

The changes in offset drift with temperatures are discussed in Reference [17]. In this context, we estimated the temperature coefficients ($\gamma_{PHMR}$) for the baseline drifts for PHMR sensors, as shown in Figure 5. It was found that the coefficient $\gamma_{PHMR}$ in CV mode was much smaller than the values in CC mode, corresponding with $\frac{\Delta R\left(T\right)}{R\left(T\right)}$ and ΔR(T), respectively (see Equations (3) and (4)). For all measured PHMR sensors, the baseline drift for all temperatures was found to be much lower (~10^−4^) compared with its intrinsic offset values. The changes in offsets for all sensors were found to be minimal over a wide temperature range from 30 °C to 90 °C (Figure 6). They fluctuated within a small range and were superposed on each other. Herein, all measurements were repeated multiple times to check the signal reproducibility of the sensors. Note that, in each case, the amplitude of baseline drift was observed to be much lower than the PHMR signal amplitude. This fact reveals that the demonstrated PHMR sensors have good thermal stability even in harsh environments.

### 4.4. Low-frequency Noise Analysis

#### 4.4.1. Decomposition of Noise Components

In order to discuss the total contributions of the different noise components for different sensors, we first estimated the effective average noise of the sensors at 100 Hz, where the white noise was prevailing compared with its *1/f* counterpart. Thus, in this context the *1/f* noise contribution was neglected. Table 1 represents the noise contribution from different noise sources, except for its *1/f* counterpart. This estimation was carried out for all PHMR sensors at two different temperatures: 30 °C and 90 °C. The sensing current was set to 1 mA. Table 1 reveals that the thermal noise contribution was more dominant in the total white noise, whereas intermixing noise was the least dominant. For the tri-mPHMR sensor at 30 °C, the dominance of thermal noise in total white noise was estimated as ~79.1%, while the least predominant intermixing noise depicted only ~3.5% contribution to total white noise. In the case of bi-cPHMR and bi-mPHMR sensors, the contributions of thermal noise are ~47.6% and ~50.7%, respectively, and the intermixing noise contributions were 1.3% and 0.03%, respectively. As reflected in Table 1, the average noise contributions for all sensors at 90 °C increased slightly from room temperature, which is expected. The lowest average noise was estimated in the bi-cPHMR sensor as only 2.29 nV/√Hz (in CC mode) due to low *R_yy_* resistance and its simple structure. Note that in this case, the estimated field sensitivity was 90 V/TA, which is much lower than that of the mPHMR sensors. Interestingly, the estimated average noise in the bi-mPHMR sensor was ~8.26 nV/√Hz, which is higher than that of the tri-mPHMR (~5.89 nV/√Hz).

**Figure 7:**
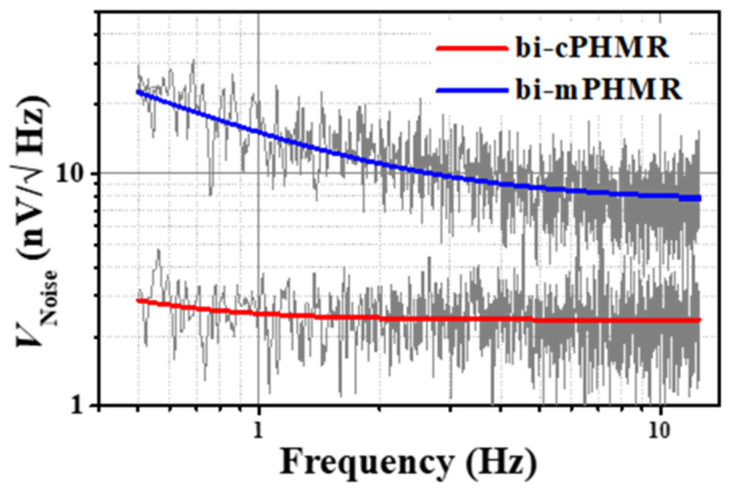
Measured voltage noise for bi-cPHMR and bi-mPHMR sensors at room temperature. Solid line depicts the fit $\mathrm{employing}\;\mathrm{the}\sqrt{a_{0}^{2}+{\left(\frac{a_{1}}{f^{\gamma }}\right)}^{2}}$ model. The sensing current was set to 1 mA. The *1/f* noise is presented in measured total average noise <15 Hz.

Contrary to low-frequency, total noise components in Equation (8) can be decomposed into white and *1/f* components as the following numerical model:

(14) $$V_{noise}^{total}=\sqrt{a_{0}^{2}+{\left(\frac{a_{1}}{f^{\gamma }}\right)}^{2}}\;\;\;\;\;\;\;\left[\frac{T}{\sqrt{Hz}}\right]\;$$

where $\;a_{0}\;\mathrm{and}\;a_{1}\;$ are the fit parameters, f is frequency, and *γ* represents the exponent of pink noise with a value close to 1 [69]. A reference low-frequency noise spectrum (below 15 Hz) for the bi-layered sensors including its fits employing the numerical model (as discussed above) is depicted in Figure 7.

In noise analysis (<15 Hz), it was found that the white noise components (thermal, intermixing, and preamplifier noise) degraded the sensor’s optimal equivalent magnetic noise by 16% at 1 Hz, 103% at 10 Hz, and more than 652% at 100 Hz, which is summarized in Table 2. Although the used preamplifier demonstrated a very low noise of 1 nV/√Hz, it was still 5 times lower than the thermal noise of the tri-mPHMR sensor (5.24 nV/√Hz). On the other hand, the degradation of sensor optimal equivalent magnetic noise was much more severe at 90 °C; estimated noise was ~1353%. A similar noise analysis was also carried out with bi-layered sensors, which is summarized in Table 3. Note that to extract the noise coefficients, we fitted the voltage noise spectra of all PHMR sensors by employing Equation (14).

#### 4.4.2. Magnetic Barkhausen Noise (MBN)

Note that as discussed above, the magnetic noise of the sensor can also be related to the stochastic behavior of the MBN in Equation (13). In order to analyze the manifestation of MBN in PHMR sensors, magnetic domains were observed by MOKE to clarify the MBN signals in the framework of discontinuous movement of the DW. For this investigation, two reference samples (bi-mPHMR and tri-mPHMR) were taken of the same sample structure size of ~1.8 × 1.8 cm^2^. The M–H loops of the employed bi-layered and tri-layered samples were measured simultaneously with domain textures observation through the in-plane magneto-optical loops.

**Figure 8:**
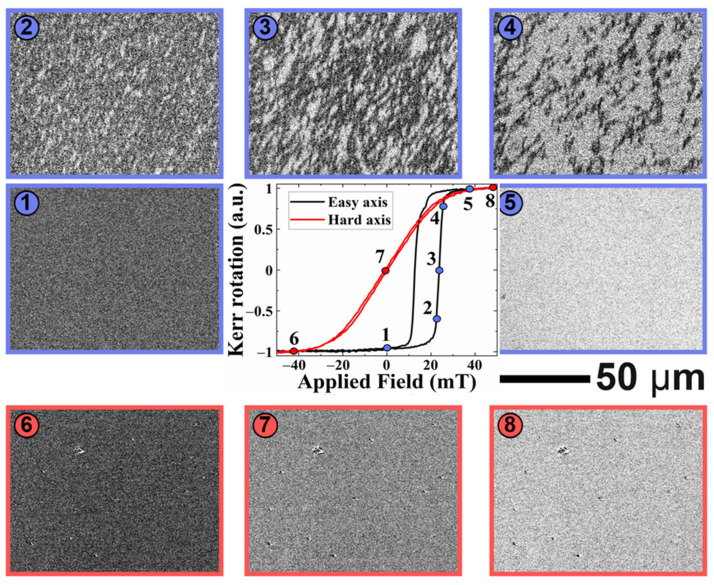
Magnetization reversal loop and magnetic domain evolution in bi-layer structure, measured by Kerr magnetometry with the external magnetic field applied along easy axis *μ*_0_*H_x_* (black curve) and along hard axis *μ*_0_*H_y_* (red curve).

Figure 8 shows the evolution of magnetic domains and magnetization reversal loop in bi-layer structures. When the external magnetic field was applied along the easy axis (black curve in Figure 8), the biased square hysteresis loop was observed. After saturating the sample by negative field (state 1 in Figure 8), application of a small reversal field resulted in nucleation of magnetic domains with opposite magnetization (state 2). The domain nucleation was followed up by the 180° magnetization domain wall motion with an increasing magnetic field until the whole area was switched (states 3 to 5). Such behavior suggests the existence of MBN in these exchanges, coupled with PHMR sensors (along the easy axis). In most cases, the contribution of MBN to total sensor noise in the magnetic sensor varied in between ~$\frac{pV}{\sqrt{Hz}}$ and a few $\frac{nV}{\sqrt{Hz}}$ [70], depending upon the sensor structures and the sensor mechanism.

On the other hand, when the external magnetic field was applied along the hard axis (red curve in Figure 8), instead of domain nucleation and domain wall movement, coherent magnetization rotation was observed: the image grey level gradually changed upon the field sweep from a negative to a positive saturation state (states 6 to 7). Thus, no MBN is expected.

**Figure 9:**
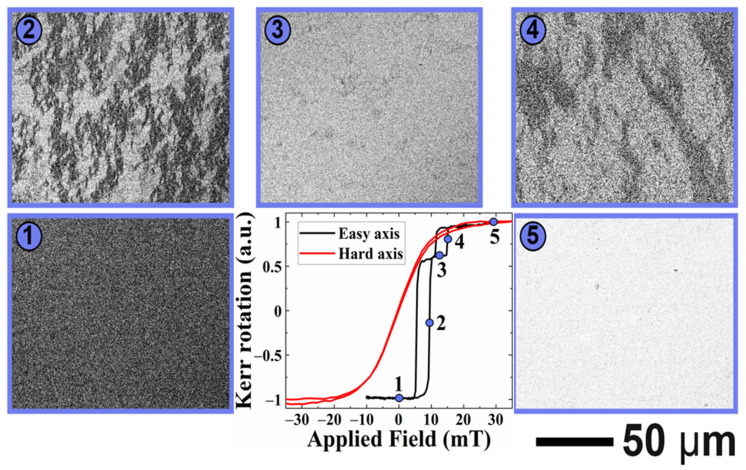
Magnetization reversal loop and magnetic domain evolution in tri-layer structure measured by Kerr magnetometry with the external magnetic field applied along easy axis (black curve) and along hard axis (red curve).

Similar behavior was also observed in tri-layer structures (see Figure 9): the nucleation and growth of magnetic domains via domain wall movement in the case of the magnetic field applied along the easy axis and coherent rotation when the field was applied along the hard-magnetic axis.

**Figure 10:**
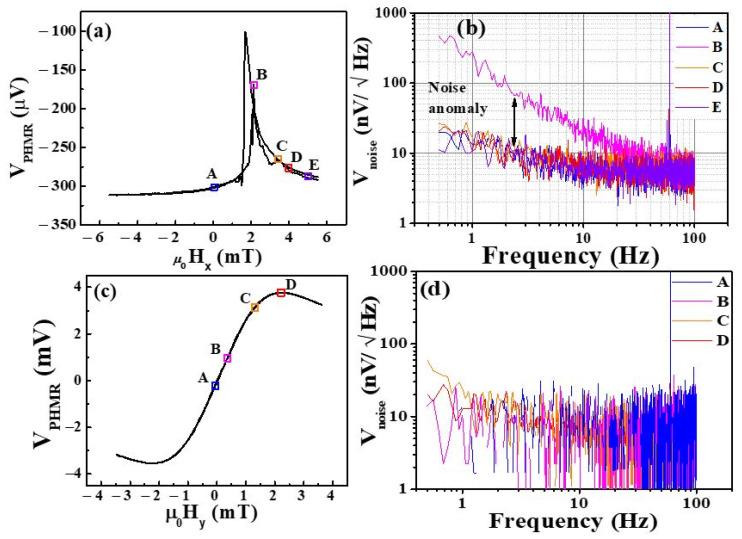
(**a**) $V_{PHMR}$-$H_{x}$ curve of the tri-mPHMR device. (**b**) Noise voltage spectra of the tri-mPHMR sensor when the external field was applied in parallel to the sensor easy axis (along *x*-axis). (**c**) $V_{PHMR}$-$H_{y}$ curve of the tri-mPHMR device. (**d**) Noise voltage spectra of the tri-mPHMR sensor when the external field was applied transversely to the sensor easy axis (along *y*-axis). All measurements were performed at room temperature, and the sensing current was set to 1 mA.

Furthermore, MBN contributions in the total average noise were also analyzed through NSDs analysis at room temperature. To estimate the MBN noise contribution in PHMR sensors, we measured the total average noise of the tri-mPHMR sensor in the frequency range of 0.5 to 100 Hz under different external field orientations. In the present study, we applied the field in two directions—(a) parallel to the easy axis denoted by *μ*_0_*H_x_* and (b) transverse to the easy axis denoted by *μ*_0_*H_y_*. Figure 10a shows the PHMR signals at 1 mA sensing current in the field range of ±6 mT. Note that the field was applied antiparallel to the exchange bias field, which resulted in the occurrence of magnetic switching peaks at 1.72 and 2.15 mT. These observed results were closely correlated with the 180° multi-domain characteristic, as revealed in Figure 8 and Figure 9. Figure 10b exhibits the voltage NSDs at the indicated *μ*_0_*H_x_* field values. Here, five field values were chosen for further evaluation. The same device sensing current of 1 mA was applied (see Figure 10a). The corresponding fields referred to here are: *μ*_0_*H_x_* = 0 mT (parallel magnetization state—A), +2.15 mT, 3.38 mT, 3.94 mT (intermediate magnetization states—B, C, D), and 4.95 mT (single magnetization state—E). At zero and 4.95 mT, it was found that both NSDs exhibited similar behavior to that in the zero field. Interestingly, in state B, an incredibly large amount of noise at small frequencies was observed. This noise was caused by the spin fluctuations between neighboring 180° domains and set a 180° angle between intra-domains, which is associated with the domain wall movements. However, for the other two intermediate field states (C and D), such a noise anomaly was not observed, which correlated with the absence of domain wall movement and the presence of only coherent magnetization rotation, i.e., effective single domain behavior. These results confirm that the voltage noise anomaly associated with NSDs at B state in parallel field configuration appeared due to the presence of MBN in the system, similar to the magnetic anomaly in a TMR sensor [71].

In next step, we estimated the MBN contributions in this tri-mPHMR sensor at 10 Hz from the vertical shift of NSDs between states Aor E and B, and the MBN noise value was ~$15\;\frac{nV}{\sqrt{Hz}}$, whereas at 1 Hz, the estimated MBN was ~$215\frac{nV}{\sqrt{Hz}}$. The extracted noise amplitudes for these ring sensors corroborate our previous report [56].

On the other hand, similar measurements were performed at different external fields; when *μ*_0_*H_y_* was transverse to the sensor easy axis (see Figure 10c), conventional *V_PHMR_* vs. *μ*_0_*H_y_* dependence was obtained. Although the amplitudes for parallel and perpendicular fields were similar, in later cases, no change in magnetic voltage noise spectrum was observed, which was in line with the magnetic domain behavior for these two orthogonal directions (see Figure 8 and Figure 9). As seen in Figure 10d, all recorded NSDs for different perpendicular fields were almost superposed on each other at low frequency. Changes in *H_y_* introduced the intermixing noise due to sensor intrinsic offsets (shift of offset noise level, as is shown in Figure 10d), which was subtracted to obtain the pure NSDs at the selected setpoints. Importantly, here all NSDs showed similar characteristics, and no voltage noise anomaly was observed. These results confirm that there was no MBN component for the conventional PHMR field sensing configuration.

#### 4.4.3. Low-Frequency Noise Model—Field Detectivity

**Figure 11:**
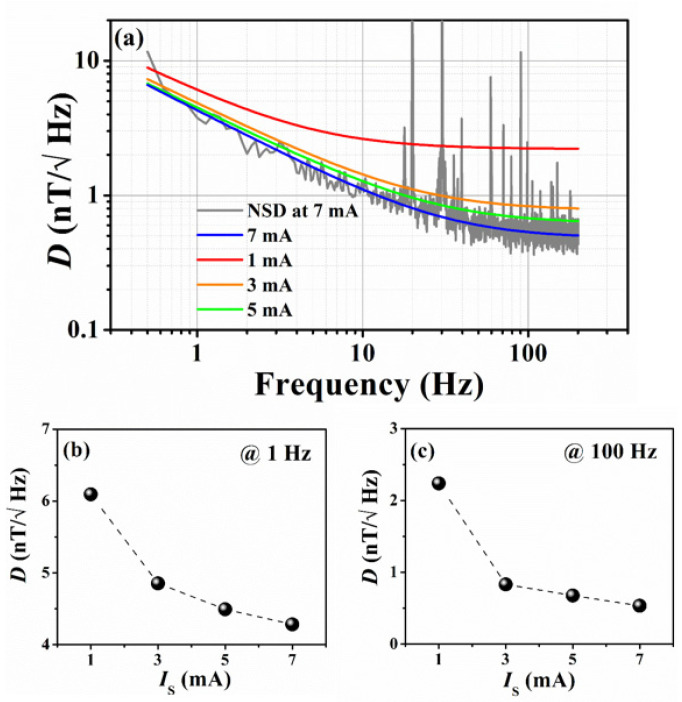
(**a**) Detectivity for tri-mPHMR sensor for different currents in CC mode; recorded room temperature NSD at 7 mA is shown in the background (gray); (**b**,**c**) exhibit change in detectivity at 1 Hz and 100 Hz, respectively (with dashed lines as a guide to the eye).

Figure 11a shows the low-frequency detectivities for different sensing currents, along with the fits according to Equation (14) for the tri-mPHMR sensor. The best detectivity was obtained at 7 mA (see blue line) for the tri-mPHMR sensor at room temperature, and the estimated D values were 521 pT/√Hz at 100 Hz, 1.3 nT/√Hz at 10 Hz, and 4.3 nT/√Hz at 1 Hz. For the tri-mPHMR sensor, the estimated fit parameters values at room temperature were $a_{0}=2.21$ and $a_{1}=5.68\;$ for 1 mA; $a_{0}=0.78$ and $a_{1}=4.79\;$ for 3 mA; $a_{0}=0.61$ and $a_{1}=4.45\;$ for 5 mA, and $\;a_{0}=0.48$ and $a_{1}=4.25\;$ for 7 mA. The estimated power coefficient *γ* was ~0.63.

The obtained detectivities for different currents for all sensors in CC mode are summarized in Table 4. It is evident from the table that for the tri-mPHMR sensor, the best *D* was observed at the maximum permissible sensing current of 7 mA at room temperature; however, for the other two sensors, the optimal sensing current for the low-frequency detectivity was found to be lower than 7 mA. Improvements in the detectivity limit in the tri-mPHMR sensor with different sensing currents for 1 Hz and 100 Hz are shown in Figure 11b,c respectively.

On the other hand, the best detectivities obtained for bi-mPHMR and bi-cPHMR sensors were 3.5 nT/√Hz at 100 Hz and 11.5 nT/√Hz at 100 Hz for the sensing currents 3 mA and 7 mA, respectively. The best fit parameters at 3 mA for bi-mPHMR sensors were $a_{0}=3.3$ and $a_{1}=31.6\;$ at 3 mA, with *γ* = 0.7, whereas for the bi-cPHMR case, the obtained fit parameters were $a_{0}=11.5$ and $a_{1}=322\;$ at 7 mA, with *γ* close to 1. It was found that the estimated fit parameters in bi-cPHMR sensors were worse due to higher *H_ex_* (~13 mT) in higher currents compared with 1 mA, but the dynamic range was the largest.

Generally, low sensing current provided similar results at low frequencies but worse results at higher frequencies, where the *1/f* noise was not so dominant. Similar characteristics were observed for the other two sensors, which are not shown here. A similar trend in sensing current optimization for a single-layered planar-Hall sensor was observed by Grosz et al. [42]. Note that in the present case, a maximum 5-fold increment in detectivity in a tri-mPHMR sensor was observed at 100 Hz, compared with its bilayer counterpart. However, at 1 Hz, the increment was within 4-fold.

**Figure 12:**
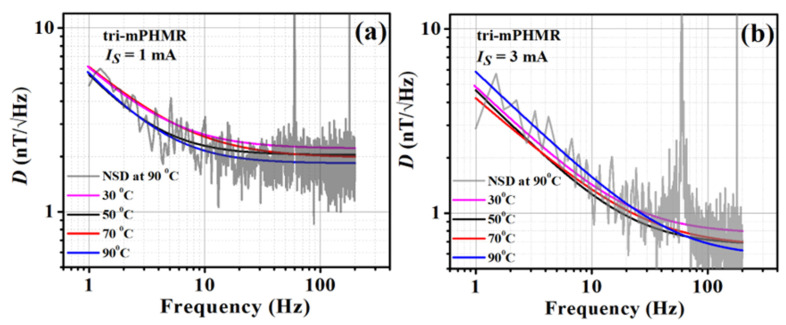
Detectivity plots at different temperatures and the fits employing Equation (14) at two different sensing currents: 1 mA (**a**) and 3 mA (**b**). Recorded NSD at 90 °C is shown in the background (gray).

Figure 12a,b shows the temperature-dependent detectivities of the tri-mPHMR sensor for 1 mA and 3 mA, respectively. As discussed earlier, it was not possible to pass a higher current >3 mA because of technical constraints. It can be seen that in both cases, the highest detectivity was achieved at 90 °C. However, with 3 mA at 90 °C, detectivity showed a higher noise value below 80 Hz. It can be seen that the best detectivity at high temperature applying 3 mA sensing current was ~680 pT/√Hz at 100 Hz, whereas, at 1 mA, the best detectivity was obtained at ~1.83 nT/√Hz at 100 Hz. However, in low-frequency noise (<10 Hz), a signature of thermal drift was observed at 3 mA with an increase in temperature, which eventually degraded the detection limit of the sensor itself below 10 Hz. Note that for very high sensing currents (>3 mA), the sensors reached their maximum threshold limit of heat dissipation and became thermally unstable, which eventually degraded the detectivity of the sensor. In addition, the sensor power consumption was also limited; thus, to achieve the best detectivity at all frequencies, it is required to set the sensing current at its optimal value of mA.

**Figure A2:**
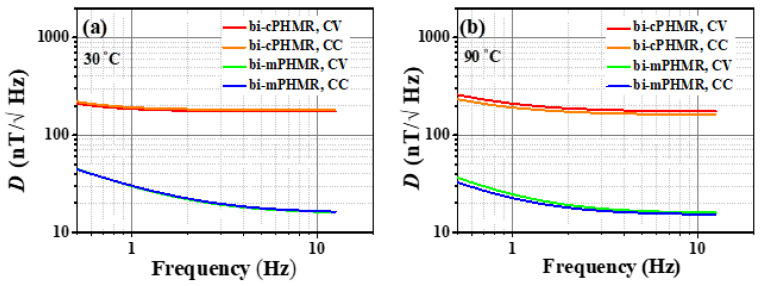
(**a**,**b**) Field detectivity for bi-layered cPHMR and mPHMR sensors at 30 °C and 90 °C. Equivalent magnetic noise, or detectivity data was fitted with Equation (14). These measurements were carried out to investigate the NSDs’ dependence on CC and CV modes.

In order to investigate the effect of different electronic load modes on NSDs, a comparative noise analysis was performed in both CC and CV modes for all PHMR sensors. Note that even with the temperature-dependent variations, no significant change was observed in the studied PHMR sensors. A comparative equivalent magnetic noise spectra (detectivity) analysis for bi-cPHMR and mPHMR sensors was recorded in both modes at 30 °C and 90 °C and is depicted in Figure A2 (see Appendix A section). It was found NSDs measured at room temperature in both electronic load modes were comparable with each other; however, at 90 °C, the detectivity in CC mode was slightly better. Similar sensitivity and noise measurements were studied additionally for two identical sensors in each case, and the reproducibility of the sensor sensitivity and detectivity was confirmed within an error bar of 10%.

It was found that the low-frequency detectivity showed a strong dependence on sensing current *I_S_*. Moreover, the obtained results confirm that the detectivity could also be improved by increasing the dimensions of the bridge. Furthermore, it can be understood that the *1/f* noise-independent part of the detectivity (the high-frequency region) was suppressed by increasing *I_S_* (see Figure 12b) and temperature, resulting in high power consumption. For low power consumption, it is required to keep the sensing current as low as possible [45].

The magnetic field detectivity of our sensors can be further improved by increasing the sensor area, optimizing the material compositions and geometry, measuring in a magnetic shield environment, and implementing the magnetic flux concentrators. For achieving better temperature dependence on detectivity based on PHMR sensors, future work is needed to find ways to reduce the temperature-independent portion from the total average noise contribution. Note that it is possible to substantially enhance the low-frequency sensor detectivity by reducing the sensor intrinsic offsets. Technically, it is possible to diminish the sensor offset using sensor a self-balancing feature to improve the sensitivity and thus the field-detectivity of the sensor [21]. Moreover, by optimizing the sensor geometry by introducing more rings, it is possible to significantly increase the sensor sensitivity, which might be a promising solution for enhancing the detection limit of the sensor. Further improvement is also possible by using a low-noise preamplifier (<1 nV/√Hz) [72], especially for the sensible noise measurements below 10 Hz. In that case, it would be possible to detect extremely low level of the voltage or current fluctuations.

## 5. Conclusions

In this study, we measured and analyzed the low-frequency noise and detectivity of cross junction and multi-ring PHMR sensors within the frequency range of 0.5 Hz to 200 Hz in an unshielded environment. To investigate the low-frequency noise and detectivity performance of the PHMRs, the sensors were measured at different temperatures and sensing current variations in a harsh environment. The spectra were measured with an external magnetic field applied in both parallel and perpendicular to the easy axis directions. The presence of Barkhausen noise was confirmed in the low-frequency noise spectrum in the case of the field along the easy axis; however, no signature of MBN was observed for the field along the hard axis, i.e., conventional PHMR configuration.

By exciting the sensor with an optimal dc current and optimizing the sensor geometry, we improved its resolution at low frequencies, exhibiting detectivity values less than 550 pT/√Hz at 100 Hz and less than 1.5 nT/√Hz at 100 Hz for the tri-mPHMR sensor. The sensors’ characterizations were conducted in a high precision, low-power (<15 mW at 7 mA) mode considering its miniatured size capable of detecting low-noise in harsh environments. Moreover, all the measured results were validated.

Our data reveal that it is more desirable to use a bilayer sensor for the larger dynamic range of ~10 mT, but a tri-layer sensor can be used for higher resolution. In order to improve the sensor detection limit further, tri-mPHMR sensors (>5 rings) and an adjustable electronic circuit with some advanced noise reduction technologies [15,16,21] can be designed next. PHMR sensors are therefore general-purpose high-performance magnetometers that can potentially satisfy most kinds of requirements for a wide range of applications. In particular, we believe that the demonstrated field resolution of the PHMR sensors in an unshielded environment may open new opportunities in the detection of biomagnetic signals, and most importantly the sensors may have industrial applications in harsh environments.

## Figures and Tables

**Table 1:** White noise decompositions at 100 Hz at 30 °C and 90 °C. The sensing current was 1 mA.

Sensor Type 30 °C	Total White Noise (nV/√Hz)	Thermal Noise (nV/√Hz)	Preamp (nV/√Hz)	Intermixing Noise (nV/√Hz)	Environmental Noise (nV/√Hz)
bi-cPHMR	2.29	1.58	1.00	0.039	1.32
bi-mPHMR	8.26	5.88	1.00	0.931	5.64
tri-mPHMR	5.89	5.24	1.00	1.10	2.24
**90 °C**	**Total White Noise** **(nV/√Hz)**	**Thermal Noise** **(nV/√Hz)**	**Preamp Noise** **(nV/√Hz)**	**Intermixing Noise** **(nV/√Hz)**	**Environment Noise** **(nV/√Hz)**
bi-cPHMR	2.50	1.79	1.00	0.039	1.43
bi-mPHMR	9.22	6.63	1.00	1.10	6.31
tri-mPHMR	6.21	5.89	1.00	1.25	1.14

**Table 2:** Total average noise decomposition for tri-mPHMR sensors at two different temperatures. The sensing current was 1 mA (fit with Equation (14)).

	30 °C			90 °C		
Frequency (Hz)	$a_{1}/$*f*^γ^, 1/f Noise Component	$a_{0},$ White Noise Component	% = $(a_{0}/$ $(a_{1}/$ *f*^γ^)) × 100	$a_{1}/$*f*^γ^, 1/f Noise Component	$a_{0},$ White Noise Component	% = $(a_{0}/$ $(a_{1}/$ *f*^γ^)) × 100
1	3.59 × 10^−5^	5.89 × 10^−6^	16%	2.57 × 10^−5^	5.50 × 10^−6^	21%
10	5.69 × 10^−6^	5.89 × 10^−6^	103%	3.23 × 10^−6^	5.50 × 10^−6^	170%
100	9.02 × 10^−7^	5.89 × 10^−6^	652%	4.07 × 10^−7^	5.50 × 10^−6^	1353%

**Table 3:** Total noise analysis and noise decompositions for bi-layered cPHMR and mPHMR sensors at room temperature. $a_{0}$: white noise component; $a_{1}/f^{\gamma }$: 1/f noise component.

	1/f Noise Component		Total White Noise (nV/√Hz)	Thermal Noise (nV/√Hz)	Environment Noise (nV/√Hz)	Intermixing Noise (nV/√Hz)	Preamplifier Noise (nV/√Hz)
	a_1_	γ					
bi-cPHMR	$\mathrm{8.7}\;\times \;10^{-7}$	0.9	2.29	1.58	1.32	0.07	1.00
bi-cPHMR	$\mathrm{1.3}\;\times \;10^{-5}$	0.7	7.87	5.88	5.05	0.92	1.00

**Table 4:** Detectivity for PHMR sensors for different sensing currents in CC mode.

Sensor Type	I_S_ (mA)	D (nT/√Hz) at 100 Hz	D (nT/√Hz) at 10 Hz	D (nT/√Hz) at 1 Hz
bi-cPHMR	1	75.1	176.1	200.5
	5	14.7	16.4	136.2
	7	11.5	15.1	322.5
bi-mPHMR	1	10.4	16.3	58.3
	3	3.5	7.1	31.8
	4	2.7	6.8	50.4
tri-mPHMR	1	2.1	2.7	6.1
	5	0.6	1.2	4.4
	7	0.52	1.1	4.3

## Data Availability

All data generated or analyzed during this work are included in this article.
